# Bridging the gap: attitudes and practices toward complementary and alternative medicine among oncology patients and healthcare professionals in Croatia

**DOI:** 10.3389/fpsyg.2025.1531111

**Published:** 2025-01-29

**Authors:** Ljerka Armano, Vanja Vasiljev, Tomislav Rukavina, Denis Juraga, Aleksandar Racz, Vanja Tešić

**Affiliations:** ^1^Faculty of Medicine, University of Rijeka, Rijeka, Croatia; ^2^University of Applied Health Sciences, Zagreb, Croatia; ^3^Teaching Institute for Public Health "Dr. Andrija Stampar", Zagreb, Croatia

**Keywords:** complementary and alternative medicine, nurses, medical doctors, attitudes, oncological patients

## Abstract

The prevalence of complementary and alternative medicine (CAM) use among oncology patients ranges from 30 to 80%, particularly higher in the United States compared to Europe. However, limited research exists on the attitudes of healthcare professionals and oncology patients toward CAM, especially within Western evidence-based medical settings. This study aims to address this gap by assessing CAM use prevalence among healthcare professionals and oncology patients and analyzing their cognitive, affective, and behavioral attitudes. Additionally, it explores the influence of sociodemographic factors and personal experiences on these attitudes. A cross-sectional survey was conducted between November 2022 and May 2023 at University Hospital Center Sisters of Mercy, Zagreb, Croatia. The study included 832 participants: 411 oncology patients and 421 healthcare professionals (100 physicians, 321 nurses/technicians). Data were collected using modified versions of the Health Belief Questionnaire (CHBQ) and Integrative Medicine Attitude Questionnaire (IMAQ). Statistical analysis included descriptive methods and tests such as Chi-square, Mann–Whitney U, Kruskal-Wallis, ANOVA, and *post hoc* Tukey tests. The results showed that 55.6% of oncology patients and 32.2% of healthcare workers had used CAM at least once. Oncology patients were more likely to use CAM than healthcare professionals, and among healthcare professionals, nurses/technicians used CAM more frequently than physicians. Significant differences in attitudes were observed based on sociodemographic factors. Positive attitudes were more common among women, older adults, individuals with lower education levels, nurses/technicians, those with longer work experience, non-oncology healthcare workers, believers, and those with lower incomes. Marital status and place of residence showed no significant effect. This study highlights a gap between cancer patients’ frequent, unsupervised CAM use and healthcare providers’ often skeptical attitudes, particularly among physicians. The findings underscore the need for targeted education for healthcare professionals, development of CAM management guidelines in oncology, and fostering open dialogue between patients and providers to optimize outcomes. Longitudinal research is recommended to explore CAM’s impact on clinical outcomes.

## 1 Introduction

### 1.1 Background

Complementary and Alternative Medicine (CAM) encompasses a broad spectrum of therapeutic approaches and practices that fall outside the boundaries of conventional medicine and are often excluded from mainstream healthcare systems. These methods have historical roots spanning centuries, with contemporary research and application demonstrating their growing relevance, particularly in the field of oncology. Recognizing their potential, numerous countries and institutions, including the World Health Organization, emphasize the importance of researching and integrating CAM into healthcare frameworks to enhance the management of chronic conditions such as cancer (World Health Organization, 2024).

In oncology, CAM is commonly adopted by patients aiming to improve their overall well-being and to address symptoms and side effects stemming from conventional cancer treatments like chemotherapy and radiation therapy. Although these standard therapies are effective, they are frequently associated with debilitating side effects, including nausea, fatigue, pain, and psychological distress. CAM offers a complementary role, providing strategies to alleviate these adverse effects and enhance patients’ quality of life. Data from the National Cancer Institute indicate that nearly 40% of cancer patients engage in CAM practices, with modalities such as herbal medicine, meditation, and manual therapies ranking among the most utilized (National Cancer Institute, 2024).

### 1.2 Statement of the problem

Despite the growing prevalence of CAM among patients, a significant barrier persists in the form of inadequate communication between patients and healthcare professionals. Many healthcare providers cite limited knowledge of CAM therapies as a critical impediment, often resulting in skepticism and a cautious approach toward their patients’ use of these modalities. This knowledge gap can influence clinical attitudes and hinder the provision of comprehensive care. Providers face a dual imperative: they must address patients expressed needs for additional therapeutic support while ensuring patient safety, particularly concerning potential adverse interactions between CAM and conventional medical treatments. These challenges are exacerbated by a lack of standardized education and robust regulatory frameworks surrounding CAM, underscoring the urgent need for an integrative healthcare paradigm that prioritizes informed decision-making and evidence-based communication (Matjuschenko et al., 2023).

Comprehensive national survey data from 2012 indicate that 33.2% of adults and 11.6% of children in the United States have utilized CAM therapies (Clarke et al., 2015). CAM utilization is especially pronounced among oncology patients, with reported usage rates varying considerably based on cancer type and demographic factors. A systematic study by Davis et al. (2012) highlights a wide range of CAM adoption among cancer patients, spanning 11–95%, while other investigations report usage rates of 30–50% (Bahall and Legall, 2017; Kwon et al., 2019; Jermini et al., 2019). Among adults, the predominant CAM modalities include natural products, deep breathing techniques, yoga, tai chi, meditation, chiropractic interventions, and massage therapy. These practices are primarily employed to address pain management needs, particularly for chronic conditions such as back, neck, and joint pain (Nahin et al., 2024).

### 1.3 Rationale

The widespread utilization of CAM highlights a growing patient inclination toward exploring alternative healthcare approaches. This increasing prevalence underscores the integration of CAM within healthcare systems globally and signals the necessity for rigorous scientific investigation to evaluate the safety, efficacy, and potential integration of these therapies into standard oncology care. CAM usage among oncology patients demonstrates substantial variation across different regions, shaped by cultural, social, and healthcare system-specific factors.

In Europe, the adoption of CAM among oncology patients is notably high, with approximately 50% of cancer patients reporting its use (Rossi et al., 2015). This trend is consistent with global patterns where CAM is often employed alongside conventional cancer therapies to enhance quality of life and mitigate treatment-related side effects (Ashrafizadeh and Rassouli, 2024). For instance, a Belgian study found that over half of cancer patients utilized natural products, with vitamins being the most consumed, followed by probiotics and herbal supplements (Schils et al., 2023). Similarly, in Germany, about 50% of cancer patients reported using CAM, primarily for improving quality of life and alleviating adverse effects of conventional treatments (Hübner et al., 2023). In Italy, the prevalence of Traditional, Complementary, Integrative, and Alternative Medicine (TCIM) among cancer patients reached 72.3%, with many initiating these therapies after receiving a cancer diagnosis (Bonucci et al., 2022). Poland reported an even higher prevalence, with 85.09% of cancer patients engaging in CAM practices such as vitamin C supplementation, green tea consumption, and prayer (Ślusarska et al., 2020).

This consistent global pattern emphasizes the critical need for comprehensive research to address the clinical and scientific gaps associated with CAM, ensuring its safe and effective incorporation into oncology care.

### 1.4 Significance

#### 1.4.1 Interest in CAM among healthcare professionals

The interest in CAM is not confined to patients; healthcare professionals also exhibit varying levels of engagement with these practices, influenced by their field of specialization, clinical experience, and personal attitudes. Although many healthcare providers express favorable views of CAM, its practical implementation remains inconsistent. This highlights the pressing need for comprehensive education, training, and professional development to facilitate evidence-based CAM integration into clinical practice. Enhancing healthcare professionals’ awareness and understanding of CAM is essential for ensuring safe and effective therapeutic applications within oncology care frameworks.

For instance, in Saudi Arabia, the prevalence of CAM use among healthcare workers reaches 97%, with attitudes significantly influencing its application (*p* = 0.007) (Al-Batanony et al., 2023). In New Zealand, healthcare professionals generally demonstrate positive attitudes toward CAM. Approximately 25% of general practitioners (GPs) incorporate CAM into their clinical practice, and 82.3% refer patients to CAM practitioners. Among physiotherapists treating pregnant patients, 48.4% use acupuncture, while 37.3% of midwives recommend CAM therapies. Notably, 26% of GPs and specialists personally utilize CAM for managing their health issues, reflecting significant professional engagement with these modalities (Liu et al., 2021). These trends illustrate that healthcare professionals’ attitudes strongly correlate with their adoption of CAM practices and underscore the necessity of targeted education to align practice with evidence-based standards.

Similar trends have been observed in Croatia, where reported CAM use among healthcare workers varies between 46.8% (Armano et al., 2017) and 82% (Vitale et al., 2014), depending on the study. The most employed CAM methods include herbal remedies and manual therapies. Research highlights distinct differences in attitudes toward CAM among healthcare professionals, with physiotherapists displaying the most positive views, while physicians tend to approach CAM with skepticism (Živčić et al., 2014). Jurković and Racz investigated CAM attitudes among healthcare students, revealing that 64.5% had either personally used or had a family member who had used CAM therapies (Jurković and Racz, 2020). These findings underline the necessity of fostering a balanced understanding of CAM’s benefits and limitations through enhanced professional education to ensure its safe and effective integration into healthcare.

#### 1.4.2 The role of attitudes in CAM adoption

Understanding healthcare professionals’ and patients’ attitudes toward CAM is essential, as these attitudes significantly influence the adoption of these therapies and their impact on treatment outcomes. Zvonarević defines attitude as “an acquired tendency to respond either positively or negatively to persons, objects, or situations outside of ourselves, or to our traits, ideas, or actions,” shaped by cognitive, behavioral, and affective components (Zvonarević, 1985). Employing this three-dimensional model facilitates a comprehensive understanding of how attitudes toward CAM are formed and how they influence decision-making in healthcare.

The cognitive component of attitude encompasses an individual’s beliefs, knowledge, or perceptions about CAM, often shaped by the available information or educational exposure (Ajzen and Fishbein, 1975). The affective component involves emotional reactions, such as affinity, skepticism, or enthusiasm toward CAM practices, which can leave a profound and lasting impression (Eagly and Chaiken, 1993). The behavioral component reflects an individual’s actions or intentions regarding CAM adoption or advocacy (Petty and Cacioppo, 1986).

As a determinant of health-related behaviors, attitudes directly influence decisions about CAM use. For instance, individuals with positive attitudes toward CAM are more inclined to adopt these methods alongside conventional treatments. Education plays a pivotal role in shaping these attitudes, particularly by influencing the cognitive component, which can lead to corresponding changes in emotional and behavioral responses. Studies indicate that favorable attitudes toward CAM are more prevalent among individuals with personal experience or those from cultures with a strong tradition of CAM acceptance. These findings offer a valuable framework for understanding the formation of attitudes and their influence on health behaviors (Eagly and Chaiken, 1993).

#### 1.4.3 Relevance to Croatia

Although global trends in CAM use are well-documented, research on CAM attitudes and practices in Croatia remains limited. This study is among the first in Croatia—and one of the few globally—to examine the perspectives of both oncology patients and their healthcare providers regarding CAM. The study seeks to illuminate current practices, attitudes, and reflections in this area, offering valuable insights to enhance patient-provider communication and support patient empowerment in treatment decision-making.

Understanding the interplay of cognitive, affective, and behavioral components in shaping attitudes toward CAM will enable healthcare strategies to be better tailored for the safe, evidence-based integration of CAM into oncology care. This approach can bridge gaps in knowledge, foster trust, and optimize patient outcomes, ultimately advancing the quality and scope of cancer care in Croatia and beyond.

The main objectives of this study include:

1. to determine the prevalence of CAM use among healthcare professionals and oncology patients.
2. to analyze the cognitive, affective, and behavioral components of their attitudes toward CAM.
3. to explore the relationship between specific sociodemographic variables, personal experiences with CAM, and expressed attitudes toward CAM.

## 2 Materials and methods

### 2.1 Study design

This study employed a cross-sectional design to examine the prevalence and attitudes toward Complementary and Alternative Medicine (CAM) among oncology patients and healthcare professionals. Data collection was conducted between November 2022 and May 2023 at the Sisters of Mercy University Hospital Centre in Zagreb, Croatia. Participants were stratified into two groups: oncology patients and healthcare professionals, with the latter further subdivided into physicians and nurses/technicians.

### 2.2 Participants

#### 2.2.1 Inclusion criteria

Oncology patients: Adults (≥18 years) diagnosed with cancer, irrespective of disease stage or type, who were receiving care at the Sisters of Mercy University Hospital Centre during the study period.

Healthcare professionals: Physicians and nurses/technicians employed at the hospital during the study period, involved either directly or indirectly in oncology care.

#### 2.2.2 Exclusion criteria

Patients or healthcare professionals unable to provide informed consent due to cognitive impairment or language barriers. Healthcare professionals not practicing at the study site during the data collection period.

### 2.3 Sample size and recruitment

The target sample size was 1,200 participants (approximately 400 oncology patients and 800 healthcare professionals). Stratified random sampling was used to ensure a proportional representation of physicians and nurses/technicians within the healthcare professional group. A total of 832 participants were included: 411 oncology patients and 421 healthcare professionals (100 physicians and 321 nurses/technicians).

### 2.4 Data collection instruments

#### 2.4.1 Questionnaires

Two tailored questionnaires were developed for oncology patients and healthcare professionals based on validated instruments:

- The CAM Health Belief Questionnaire (CHBQ): Modified to assess cognitive, affective, and behavioral components of attitudes toward CAM.
- The Integrative Medicine Attitude Questionnaire (IMAQ): Adapted to capture participants’ perceptions of CAM integration with conventional medicine.

Each questionnaire comprised three sections:

- Demographics: Including age, gender, marital status, education level, income, religious affiliation, and professional experience (for healthcare professionals).
- Personal Experience with CAM: Frequency of CAM use, reasons for use, and awareness of CAM practices.
- Attitudes toward CAM: Evaluated using Likert-scale items covering cognitive, affective, and behavioral dimensions.

### 2.5 Procedure

#### 2.5.1 Recruitment

Eligible participants were approached during their hospital visits or work shifts. Oncology patients were recruited by trained researchers during outpatient or inpatient visits, while healthcare professionals were recruited via departmental meetings and workplace announcements. All participants provided written informed consent prior to participation.

#### 2.5.2 Survey administration

Surveys were administered face-to-face to minimize nonresponse and ensure clarity. Trained interviewers assisted participants in completing the questionnaires, ensuring consistency and accuracy. On average, survey completion required 20–30 min.

#### 2.5.3 Data management

Data was anonymized and entered into a secure database by trained data entry personnel. Responses were double-checked for accuracy and completeness. Identifying information was removed to maintain participant confidentiality.

### 2.6 Statistical analysis

Descriptive and inferential statistical methods were applied:

- Descriptive statistics: Frequencies, percentages, means, and standard deviations were used to summarize demographic characteristics and CAM usage patterns.

Inferential statistics:

- Comparisons: Chi-square, Mann–Whitney U, and Kruskal-Wallis tests were used for group comparisons.
- Correlations: Spearman’s correlation coefficient was calculated to assess relationships between variables.
- Analysis of Variance (ANOVA): Conducted to compare attitudes across subgroups, with *post hoc* Tukey tests applied where appropriate.

Normality of data distribution was tested using Kolmogorov–Smirnov and Shapiro–Wilk tests. Statistical significance was set at *p* < 0.05. All analyses were performed using IBM SPSS Statistics, Version 26.0.

### 2.7 Ethical considerations

Ethical approval was obtained from the Ethics Committee of the Sisters of Mercy University Hospital Center. The study adhered to the principles of the Declaration of Helsinki, Nuremberg Code, and Croatian legislation on patient rights and data protection. Participants were assured of their anonymity and the confidentiality of their data. Written informed consent was obtained from all participants, who were free to withdraw from the study at any time.

### 2.8 Questionnaires

Two tailored questionnaires were used: one for healthcare professionals and one for oncology patients. These were developed specifically for this study with minor modifications to previously validated instruments, namely the CAM Health Belief Questionnaire (CHBQ) (Lie and Boker, 2004) and the Integrative Medicine Attitude Questionnaire (IMAQ) (Schneider et al., 2003).

#### 2.8.1 Pilot testing

Permission must be obtained for the use of copyrighted material from other sources (including the web). Please note that it is compulsory to follow the figure instructions.

#### 2.8.2 Structure of the questionnaires

##### 2.8.2.1 Demographic information

For **oncology patients**, the demographic section included eight questions covering: gender, age, marital status, education level, place of residence, religious affiliation, income level, and self-assessed health status.

For **healthcare professionals**, the demographic section included 11 questions: years of professional experience, specific profession, and whether they worked directly in oncology.

##### 2.8.2.2 Attitudes about CAM

The second section focused on assessing attitudes regarding CAM, including:

- Integration of CAM with conventional medicine.
- Incorporation of CAM into medical education.
- Perceived efficacy of CAM therapies.
- Reasons for CAM usage.
- Evaluation of therapeutic effectiveness of individual techniques.

##### 2.8.2.3 Personal experience with CAM

The third section explored personal experiences with CAM, including:

- History of CAM usage.
- Frequency of use.
- Reasons for utilization.
- Expectations and perceived benefits.
- Adverse effects, if any.
- Communication with medical professionals about CAM use.
- Sources of information.
- Satisfaction with CAM practitioners’ services.
- The financial burden associated with CAM therapies.

This comprehensive design facilitated a detailed exploration of oncology patients’ and healthcare professionals’ sociodemographic determinants, attitudes, and personal experiences with CAM, providing critical insights into its usage and perceptions.

### 2.9 Data processing methods

During statistical analysis, both descriptive and inferential statistical methods were employed. Descriptive analysis involves presenting data in tables using absolute frequencies, percentages, and measures of central tendency, including the arithmetic mean, standard deviation, and minimum and maximum values. Spearman’s correlation coefficient (r) was calculated on a scale of −1 to 1 to assess relationships between variables and categories, indicating the direction (positive or negative) and intensity of correlations. Based on the data distribution, parametric or non-parametric statistical methods were applied, with normality tested using the Kolmogorov–Smirnov and Shapiro–Wilk tests. Statistical significance was evaluated through the Chi-square, Mann–Whitney U, and Kruskal-Wallis tests. Differences among the three groups were assessed using ANOVA and *post hoc* analysis with the Tukey test.

The significance of all tests during testing was set at 5%, representing a confidence level of 95%. The IBM Corp. statistical program was used for statistical processing. Released 2019. IBM SPSS Statistics for Windows, Version 26.0. Armonk, NY: IBM Corp.

## 3 Materials and methods

### 3.1 Sociodemographic characteristics of the respondents

The study included 832 participants, with 411 oncology patients and 421 healthcare professionals (100 physicians and 321 nurses/technicians). The gender distribution showed a predominance of female respondents (70.6%), with women making up 57.4% of oncology patients and 88.2% of nurses/technicians, compared to 68% of physicians. This highlights a gender imbalance in the healthcare professional strata, particularly among nursing staff, which may influence attitudes toward CAM given prior research linking gender with CAM perceptions. Age distribution revealed that most oncology patients were over 60 years old (44.3%), whereas healthcare professionals were predominantly in younger age groups, with 26.5% of nurses and technicians aged 21–30 years. This difference in age distribution may partly explain variations in CAM attitudes and usage, as older individuals often report higher CAM engagement (Tables 1–8).

**Table 1:** Descriptive data of respondents (sex, age, marital status, education level, place of residence, religious affiliation, socioeconomic status, length of employment, employment in oncology).

		Substrata (N)			
		Physicians	Nurses	Patients	Total
Sex	Male	32	38	175	245
	Female	68	283	236	587
	Total	100	321	411	832
Age	<20 y	0	0	1	1
	20–30 y	17	85	5	107
	31–40 y	26	80	12	118
	41–50 y	32	84	90	206
	51–60 y	25	64	121	210
	>60 y	0	8	182	190
	Total	100	321	411	832
Marital status	Married/in a relationship	72	212	301	585
	Single	28	109	110	247
	Total	100	321	411	832
Education level	El. school	0	0	9	9
	Sec. school	0	86	252	338
	Bach. degree	0	124	61	185
	Msn. degree	100	111	89	300
	Total	100	321	411	832
Place of residence	Urban area	100	217	283	600
	Rural area	0	104	128	232
	Total	100	321	411	832
Religious affiliation	Religious	84	273	321	678
	Agnostic	16	40	62	118
	Atheist	0	8	28	36
	Total	100	321	411	832
Income level	<1,000 €	0	31	207	238
	Cca 1,000 €	3	205	104	312
	>1,000 €	97	85	100	282
	Total	100	321	411	832
Work experience	<5 y	16	51	0	67
	5–15 y	29	76	0	105
	16–25 y	34	80	0	114
	26–35 y	21	78	0	99
	>35 y	0	36	0	36
	Total	100	321	0	421
Employment in oncology	Yes	56	72	0	128
	No	44	242	0	286
	Total	100	314	0	414

**Table 2:** Level of agreement with statements “*Do you currently use, or have you previously used, any other remedies or methods for treating illness besides those prescribed by your doctor?*” and “*How often do you currently (or have you previously) use/practice CAM?*”.

		Strata					
		Patients		HCW		Total	
		N	%	N	%	N	%
Do you currently use, or have you previously used, any other remedies or methods for treating illness besides those prescribed by your doctor?	Yes	228	55.6	136	32.2	364	43.8
	No	182	44.4	284	67.9	468	56.3
	Total	410	100	422	100	832	100
How often do you currently (or have you previously) use/practice CAM?	Never	176	42.9	239	56.6	415	49.9
	Rarely	23	5.6	61	14.5	84	10.1
	Occasionally	92	22.4	92	21.8	184	22.1
	Once a week	7	1.7	11	2.6	18	2.2
	Every day	112	27.3	19	4.5	131	15.7
	Total	410	100	422	100	832	100

**Table 3:** Significance of differences between strata and substrata using Tukey’s test for the statements “*Do you currently use, or have you previously used, any other remedies or methods for treating illness besides those prescribed by your doctor?*” and “*How often do you currently (or have you previously) use/practice CAM*?”.

	(I) Strata	(J) Substrata	Mean difference (I-J)	Std. Error	Sig.	95% Confidence interval	
						Lower bound	Upper bound
Do you currently use, or have you previously used, any other remedies or methods for treating illness besides those prescribed by your doctor?	Physicians	Nurses	0.277*	0.055	0.000	0.15	0.41
		Patients	0.457*	0.054	0.000	0.33	0.58
	Nurses	Physicians	−0.277*	0.055	0.000	−0.41	−0.15
		Patients	0.180*	0.036	0.000	0.10	0.26
	Patients	Physicians	−0.457*	0.054	0.000	−0.58	−0.33
		Nurses	−0.180*	0.036	0.000	−0.26	−0.10
How often do you currently (or have you previously) use/practice CAM?	Physicians	Nurses	−0.680^*^	0.113	0.000	−0.94	−0.42
		Patients	−0.827*	0.160	0.000	−1.20	−0.45
	Nurses	Physicians	−1.435*	0.156	0.000	−1.80	−1.07
		Patients	0.827*	0.160	0.000	0.45	1.20
	Patients	Physicians	−0.607*	0.104	0.000	−0.85	−0.36
		Nurses	1.435*	0.156	0.000	1.07	1.80

**Table 4:** Comparison based on the question “*Do you currently use, or have you previously used, any other remedies or methods for treating illness besides those prescribed by your doctor?*”.

		Do you currently use, or have you previously used, any other remedies or methods for treating illness besides those prescribed by your doctor?						p*
		Yes		No		Total		
		N	%	N	%	N	%	
Strata	Patients	228	62%	182	39.2%	410	49.3%	*χ*^2^ = 42.431 df = 1 *p* = **0.000**
	HCW	140	38%	282	60.8%	422	50.7%	
	Total	368	100%	464	100%	832	100%	
Sex	Male	88	23.9%	157	33.8%	245	29.4%	*χ*^2^ = 9.727 df = 1 *p* = **0.002**
	Female	280	76.1%	307	66.2%	587	70.6%	
	Total	368	100%	464	100%	832	100%	
Age	<20	1	0.3%	0	0%	1	0.1%	*χ*^2^ = 20.783 df = 5 *p* = **0.001**
	20–30 y	28	7.6%	79	17%	107	12.9%	
	31–40 y	47	12.8%	71	15.3%	118	14.2%	
	41–50 y	100	27.2%	106	22.8%	206	24.8%	
	51–60 y	97	26.4%	113	24.4%	210	25.2%	
	>60 y	95	25.8%	95	20.5%	190	22.8%	
	Total	368	100%	464	100%	832	100%	
Marital status	Married/in a relationship	267	72.6%	318	68.5%	585	70.3%	*χ*^2^ = 1.589 df = 1 *p* = 0.208
	Single/divorced/widowed	101	27.4%	146	31.5%	247	29.7%	
	Total	368	100%	464	100%	832	100%	
Educational level	El. school	2	0.5%	7	1.5%	9	1.1%	*χ*^2^ = 13.455 df = 3 *p* = **0.004**
	Sec. school	166	45.1%	172	37.1%	338	40.6%	
	Bach. degree	90	24.5%	95	20.5%	185	22.2%	
	Msn. degree	110	29.9%	190	40.9%	300	36.1%	
	Total	368	100%	464	100%	832	100%	
Substrata	Physicians	10	2.7%	90	19.4%	100	12%	*χ*^2^ = 71.616 df = 2 *p* = **0.000**
	Nurses	129	35.1%	192	41.4%	321	38.6%	
	Patients	229	62.2%	182	39.2%	411	49.4%	
	Total	368	100%	464	100%	832	100%	
Work experience	<5 y	14	10%	53	18.8%	67	15.9%	*χ*^2^ = 8.456 df = 4 *p* = 0.076
	5–15 y	33	23.6%	72	25.5%	105	24.9%	
	16–25 y	41	29.3%	73	25.9%	114	27%	
	26–35 y	35	25%	65	23%	100	23.7%	
	>35 y	17	12.1%	19	6.7%	36	8.5%	
	Total	140	100%	282	100%	422	100%	
Employment in oncology	Yes	32	23.2%	95	34.4%	127	30.7%	*χ*^2^ = 5.458 df = 1 *p* = **0.019**
	No	106	76.8%	181	65.6%	287	69.3%	
	Total	138	100%	276	100%	414	100%	
Area of residence	Urban area	264	71.7%	336	72.4%	600	72.1%	*χ*^2^ = 0.046 df = 1 *p* = 0.829
	Rural area	104	28.3%	128	27.6%	232	27.9%	
	Total	368	100%	464	100%	832	100%	
Religious affiliation	Religious	307	83.4%	371	80%	678	81.5%	*χ*^2^ = 1.645 df = 2 *p* = 0.439
	Agnostic	47	12.8%	71	15.3%	118	14.2%	
	Atheist	14	3.8%	22	4.7%	36	4.3%	
	Total	368	100%	464	100%	832	100%	
Income level	<1,000 €	123	33.4%	115	24.8%	238	28.6%	*χ*^2^ = 18.794 df = 2 *p* = **0.000**
	1,000 €	149	40.5%	163	35.1%	312	37.5%	
	>1,000 €	96	26.1%	186	40.1%	282	33.9%	
	Total	368	100%	464	100%	832	100%	
I consider my health to be	Very poor	12	3.3%	1	0.2%	13	1.6%	
	Poor	20	5.4%	42	9.1%	62	7.5%	*χ*^2^ = 18.802 df = 4 *p* = **0.001**
	Neither good nor poor	110	29.9%	152	32.8%	262	31.5%	
	Good	185	50.3%	206	44.4%	391	47%	
	Very good	41	11.1%	63	13.6%	104	12.5%	
	Total	368	100%	464	100%	832	100%	

*p* = 0.000 is typically rounded from a very small *p*-value (e.g., <0.001) and does not literally mean zero. It indicates a statistically significant result at conventional thresholds.

**Table 5:** Comparison based on the question “*How often do you currently (or have you previously) use/practice CAM?*”.

		How often do you currently (or have you previously) use/practice CAM?				p*
		Rarely/occasionally		Once a week/every day		
		N	%	N	%	
Strata	Patients	115	42.9%	119	79.9%	*χ*^2^ = 53.106 df = 1 *p* = **0.000**
	HCW	153	57.1%	30	20.1%	
	Total	268	100%	149	100%	
Sex	Male	52	19.4%	38	25.5%	*χ*^2^ = 2.106 df = 1 *p* = 0.147
	Female	216	80.6%	111	74.5%	
	Total	268	100%	149	100%	
Age	<20	0	0.0%	1	0.7%	*χ*^2^ = 15.245 df = 5 *p* = **0.009**
	20–30 y	32	11.9%	4	2.7%	
	31–40 y	39	14.6%	15	10.1%	
	41–50 y	70	26.1%	48	32.2%	
	51–60 y	66	24.6%	45	30.2%	
	>60 y	61	22.8%	36	24.2%	
	Total	268	100%	149	100%	
Marital status	Married/in a relationship	187	69.8%	110	73.8%	*χ*^2^ = 0.766 df = 1 *p* = 0.381
	Single/divorced/widowed	81	30.2%	39	26.2%	
	Total	268	100%	149	100%	
Educational level	El. school	1	0.4%	1	0.7%	*χ*^2^ = 12.669 df = 3 *p* = **0.005**
	Sec. school	94	35.1%	78	52.3%	
	Bach. degree	75	28%	34	22.8%	
	Msn. degree	98	36.6%	36	24.2%	
	Total	268	100%	149	100%	
Substrata	Physicians	10	3.7%	2	1.3%	*χ*^2^ = 53.106 df = 2 *p* < **0.001**
	Nurses	143	53.4%	28	18.8%	
	Patients	115	42.9%	119	79.9%	
	Total	268	100%	149	100%	
Work experience	<5 y	14	9.2%	2	6.7%	*χ*^2^ = 7.639 df = 4 *p* = 0.106
	5–15 y	40	26.1%	4	13.3%	
	16–25 y	45	29.4%	7	23.3%	
	26–35 y	35	22.9%	14	46.7%	
	>35 y	19	12.4%	3	10%	
	Total	153	100%	30	100%	
Employment in oncology	Yes	37	24.7%	5	17.2%	*χ*^2^ = 0.746 df = 1 *p* = 0.388
	No	113	75.3%	24	82.8%	
	Total	150	100%	29	100%	
Area of residence	Urban area	181	67.5%	115	77.2%	*χ*^2^ = 4.324 df = 1 *p* = **0.038**
	Rural area	87	32.5%	34	22.8%	
	Total	268	100%	149	100%	
Religious affiliation	Religious	231	86.2%	120	80.5%	*χ*^2^ = 2.309 df = 2 *p* = 0.315
	Agnostic	29	10.8%	23	15.4%	
	Atheist	8	3%	6	4%	
	Total	268	100%	149	100%	
Income level	<1,000 €	76	28.4%	52	34.9%	*χ*^2^ = 2.386 df = 2 *p* = 0.303
	1,000 €	115	42.9%	54	36.2%	
	>1,000 €	77	28.7%	43	28.9%	
	Total	268	100%	149	100%	
I consider my health to be	Very poor	5	1.9%	7	4.7%	*χ*^2^ = 6.931 df = 4 *p* = 0.140
	Poor	11	4.1%	9	6%	
	Neither good nor poor	86	32.1%	37	24.8%	
	Good	131	48.9%	82	55%	
	Very good	35	13.1%	14	9.4%	
	Total	268	100%	149	100%	

*p* = 0.000 is typically rounded from a very small *p*-value (e.g., <0.001) and does not literally mean zero. It indicates a statistically significant result at conventional thresholds.

**Table 6:** Significance of differences between strata and substrata using Tukey’s test.

	(I) Substrata	(J) Substrata	Mean difference (I-J)	Std. Error	Sig.	95% Confidence interval	
						Lower bound	Upper bound
I have personally used a CAM method at least once in my life.	Physicians	Nurses	−1.305^*^	0.172	0.000	−1.71	−0.90
		Patients	−1.349^*^	0.168	0.000	−1.74	−0.96
	Nurses	Physicians	1.305^*^	0.172	0.000	0.90	1.71
		Patients	−0.044	0.112	0.919	−0.31	0.22
	Patients	Physicians	1.349^*^	0.168	0.000	0.96	1.74
		Nurses	0.044	0.112	0.919	−0.22	0.31
A family member or someone close to me has used a CAM method at least once in their life.	Physicians	Nurses	−0.956^*^	0.152	0.000	−1.31	−0.60
		Patients	−0.579^*^	0.148	0.000	−0.93	−0.23
	Nurses	Physicians	0.956^*^	0.152	0.000	0.60	1.31
		Patients	0.377^*^	0.099	0.000	0.14	0.61
	Patients	Physicians	0.579^*^	0.148	0.000	0.23	0.93
		Nurses	−0.377*	0.099	0.000	−0.61	−0.14
During treatment, I encountered at least one patient who used a CAM method.	Physicians	Nurses	0.004	0.134	1.000	−0.31	0.32
		Patients	0.297	0.130	0.058	−0.01	0.60
	Nurses	Physicians	−0.004	0.134	1.000	−0.32	0.31
		Patients	0.293^*^	0.087	0.002	0.09	0.50
	Patients	Physicians	−0.297	0.130	0.058	−0.60	0.01
		Nurses	−0.293^*^	0.087	0.002	−0.50	−0.09
I would gladly use CAM methods but cannot afford them due to financial reasons.	Physicians	Nurses	−1.285^*^	0.136	0.000	−1.60	−0.97
		Patients	−1.355^*^	0.132	0.000	−1.67	−1.05
	Nurses	Physicians	1.285^*^	0.136	0.000	0.97	1.60
		Patients	−0.070	0.088	0.706	−0.28	0.14
	Patients	Physicians	1.355^*^	0.132	0.000	1.05	1.67
		Nurses	0.070	0.088	0.706	−0.14	0.28
I am seriously considering seeking help from a CAM therapist.	Physicians	Nurses	−1.366^*^	0.136	0.000	−1.69	−1.05
		Patients	−1.455^*^	0.133	0.000	−1.77	−1.14
	Nurses	Physicians	1.366^*^	0.136	0.000	1.05	1.69
		Patients	−0.089	0.089	0.575	−0.30	0.12
	Patients	Physicians	1.455^*^	0.133	0.000	1.14	1.77
		Nurses	0.089	0.089	0.575	−0.12	0.30

**Table 7:** Level of agreement with the statement “*Although they do not talk about it, patients use some form of CAM therapies in practice*” by respondent groups.

		Strata					
		Patients		HCW		Total	
		N	%	N	%	N	%
Although they do not talk about it, patients use some form of CAM therapies in practice	Strongly disagree	10	2.4	17	4.0	27	3.2
	Mostly disagree	31	7.6	48	11.4	79	9.5
	Neither agree nor disagree	101	24.6	139	32.9	240	28.8
	Mostly agree	181	44.1	138	32.7	319	38.3
	Strongly agree	87	21.2	80	19	167	20.1
	Total	410	100	422	100	832	100

**Table 8:** Significance of differences between groups and subgroups using Tukey’s test.

	(I) Substrata	(J) Substrata	Mean difference (I-J)	Std. Error	Sig.	95% Confidence interval	
						Lower bound	Upper bound
Although they do not talk about it, patients use some form of CAM therapies in practice	Physicians	Nurses	−0.680^*^	0.113	0.000	−0.94	−0.42
		Patients	−0.755^*^	0.110	0.000	−1.01	−0.50
	Nurses	Physicians	0.680^*^	0.113	0.000	0.42	0.94
		Patients	−0.075	0.073	0.564	−0.25	0.10
	Patients	Physicians	0.755^*^	0.110	0.000	0.50	1.01
		Nurses	0.075	0.073	0.564	−0.10	0.25

### 3.2 Widespread application of certain methods and techniques from the spectrum of CAM and integrative medicine among healthcare workers and oncology patients

The analysis demonstrated that 55.6% of oncology patients and 32.2% of healthcare professionals had used CAM at least once, with statistically significant differences between these groups (*p* < 0.05). Notably, 27.3% of patients reported using CAM daily, compared to only 4.5% of healthcare professionals, further emphasizing the higher reliance on CAM among patients. These findings highlight a stronger inclination among patients to integrate CAM into their health routines, with a notable segment of the patient population engaging in daily CAM practices. In contrast, HCWs exhibit a more reserved approach, with a majority either rarely using or completely abstaining from CAM. This divergence underscores the differing perspectives and practices between these two groups, suggesting the need for further investigation into the factors driving CAM adoption and its role in healthcare settings.

The analysis of CAM usage among patients, nurses, and physicians reveals significant differences in engagement. Patients report the highest use of CAM, with a statistically significant higher likelihood compared to both nurses (mean difference: 0.180, *p* < 0.001) and physicians (mean difference: 0.457, *p* < 0.001). Nurses also engage more with CAM than physicians (mean difference: 0.277, *p* < 0.001).

In terms of frequency, patients practice CAM significantly more often than nurses (mean difference: 1.435, *p* < 0.001) and physicians (mean difference: 0.827, *p* < 0.001). Nurses also report higher frequencies than physicians (mean difference: 0.680, *p* < 0.001).

These findings highlight a clear gradient in CAM engagement, with patients being the most frequent users, followed by nurses and then physicians. The data underscores the importance of improving awareness and education about CAM among healthcare professionals to better align their practices with patient preferences and support informed decision-making.

### 3.3 Personal experience of applying CAM

The analysis reveals significant differences in CAM use across demographic and professional groups. Patients reported substantially higher CAM use (62%) compared to healthcare workers (38%) (*p* < 0.001), with nurses (35.1%) being more frequent users than physicians (2.7%). Women were significantly more likely to use CAM (76.1%) than men (23.9%) (*p* = 0.002), and middle-aged individuals (41–60 years) showed the highest prevalence of CAM use (*p* = 0.001).

Higher education and lower income were also associated with greater CAM use, with those holding a master’s degree (29.9%) and earning less than €1,000 (33.4%) being the most frequent users (*p* = 0.004, *p* < 0.001). Individuals with “poor” or “neither good nor poor” health reported higher CAM engagement compared to those in “good” or “very good” health (*p* = 0.001).

These findings underscore that CAM use is more prevalent among patients, women, and those with higher education or perceived health challenges, highlighting the need for tailored healthcare strategies to ensure informed and safe CAM integration.

The analysis revealed significant differences in the frequency of CAM use among patients and healthcare workers. Patients were significantly more likely to use CAM regularly (79.9%) compared to healthcare workers (20.1%) (*p* < 0.001). Frequent CAM use was most common among individuals aged 41–60 years and those with secondary education (52.3%) (*p* = 0.009, *p* = 0.005). Patients reported the highest frequent use (79.9%), followed by nurses (18.8%) and physicians (1.3%) (*p* < 0.001). Urban residents were more likely to use CAM frequently (77.2%) than rural residents (22.8%) (*p* = 0.038). These results emphasize the need to consider demographic and professional differences in CAM integration efforts.

The analysis reveals significant differences in CAM-related experiences and attitudes among patients, nurses, and physicians. Patients reported the highest personal use of CAM methods compared to physicians (mean difference: 1.349, *p* < 0.001) and nurses (mean difference: 0.044, *p* = 0.919). Similarly, family or close connections using CAM were more commonly reported by nurses and patients than by physicians (mean difference: 0.956 and 0.579, *p* < 0.001).

While financial constraints did not significantly impact patients’ likelihood of using CAM compared to nurses (mean difference: 0.070, *p* = 0.706), both groups reported substantially higher interest in seeking CAM therapies than physicians (mean difference: 1.285–1.455, *p* < 0.001). These findings underline the varying perceptions and experiences of CAM among different professional groups and patient populations, highlighting the need for tailored educational and integrative approaches.

### 3.4 The three-dimensional component of attitude

The data indicates a notable consensus among both patients and healthcare workers (HCWs) that patients often use some form of CAM therapies without discussing it openly. The most significant finding is the broad agreement among both patients and healthcare workers (HCWs) that CAM therapies are used by patients but often not openly discussed. Specifically, 65.3% of patients and 51.7% of HCWs either “mostly agree” or “strongly agree” with this statement, indicating that the use of CAM is widely perceived as a common, yet under-communicated practice. The behavioral analysis revealed that healthcare professionals, particularly physicians, rarely discuss CAM with patients, with only 14.5% of professionals reporting frequent CAM-related discussions. This contrasts sharply with patients’ high CAM usage rates, underscoring a critical gap in communication and guidance. This highlights the need for better communication between patients and HCWs about CAM use to ensure its safe and effective integration into healthcare.

The key finding from this data is the significant difference in perceptions between physicians and patients, as well as between physicians and nurses, regarding the use of CAM therapies by patients. Physicians are less likely to agree that patients use CAM therapies than both nurses (mean difference = −0.680, *p* < 0.001) and patients (mean difference = −0.755, *p* < 0.001). Conversely, there is no significant difference in perceptions between nurses and patients (mean difference = −0.075, *p* = 0.564). This suggests that nurses and patients share a more aligned understanding of CAM usage, while physicians are comparatively less aware or less inclined to acknowledge the prevalence of CAM use among patients.

### 3.5 Attitudes about the general reasons for using certain methods and techniques from the spectrum of cam and integrative medicine among health workers who care for oncology patients

In the following, an analysis of the degree of acceptance of claims about the reasons for using CAM is shown in percentages (Table 9), and their analysis is performed using the Tukey test (Table 10), which gives us statistical significance between and within groups.

**Table 9:** Level of agreement with statements on reasons for using cam between groups.

		Strata					
		Patients		Patients		Patients	
		N	%	N	%	N	%
Conventional medicine is overly based on technology.	Strongly disagree	24	5.9	68	16.1	92	11.1
	Mostly disagree	64	15.6	74	17.5	138	16.6
	Neither agree nor disagree	188	45.9	160	37.9	348	41.8
	Mostly agree	107	26.1	85	20.1	192	23.1
	Strongly agree	27	6.6	35	8.3	62	7.5
	Total	410	100	422	100	832	100
Conventional medicine does not treat the person as a whole.	Strongly disagree	27	6.6	68	16.1	95	11.4
	Mostly disagree	48	11.7	75	17.8	123	14.8
	Neither agree nor disagree	162	39.5	118	28	280	33.7
	Mostly agree	125	30.5	101	23.9	226	27.2
	Strongly agree	48	11.7	60	14.2	108	13
	Total	410	100	422	100	832	100
Dissatisfaction with the therapeutic capabilities of conventional medicine (treatment outcomes, drug side effects, helplessness in terminal stages of disease).	Strongly disagree	43	10.5	18	4.3	61	7.3
	Mostly disagree	41	10	33	7.8	74	8.9
	Neither agree nor disagree	162	39.5	144	34.1	306	36.8
	Mostly agree	121	29.5	141	33.4	262	31.5
	Strongly agree	43	10.5	86	20.4	129	15.5
	Total	410	100	422	100	832	100
Dissatisfaction with the attitude of doctors and healthcare workers in conventional medicine toward patients.	Strongly disagree	67	16.3	27	6.4	94	11.3
	Mostly disagree	89	21.7	60	14.2	149	17.9
	Neither agree nor disagree	174	42.4	153	36.3	327	39.3
	Mostly agree	53	12.9	112	26.5	165	19.8
	Strongly agree	27	6.6	70	16.6	97	11.7
	Total	410	100	422	100	832	100
Dissatisfaction with the healthcare system (waiting lists, high costs, lack of all medications covered by the Croatian Health Insurance Fund…).	Strongly disagree	28	6.8	21	5	49	5.9
	Mostly disagree	57	13.9	46	10.9	103	12.4
	Neither agree nor disagree	168	41.0	132	31.3	300	36.1
	Mostly agree	115	28	128	30.3	243	29.2
	Strongly agree	42	10.2	95	22.5	137	16.5
	Total	410	100	422	100	832	100
The belief that CAM will help where conventional medicine can no longer assist.	Strongly disagree	12	2.9	10	2.4	22	2.6
	Mostly disagree	19	4.6	30	7.1	49	5.9
	Neither agree nor disagree	95	23.2	123	29.1	218	26.2
	Mostly agree	214	52.2	145	34.4	359	43.1
	Strongly agree	70	17.1	114	27	184	22.1
	Total	410	100	422	100	832	100
Fear of side effects from drugs and therapeutic procedures.	Strongly disagree	20	4.9	14	3.3	34	4.1%
	Mostly disagree	33	8	41	9.7	74	8.9
	Neither agree nor disagree	113	27.6	177	41.9	290	34.9
	Mostly agree	189	46.1	128	30.3	317	38.1
	Strongly agree	55	13.4	62	14.7	117	14.1
	Total	410	100	422	100	832	100
Desire for the patient to take a more active role in their treatment.	Strongly disagree	10	2.4	7	1.7	17	2
	Mostly disagree	24	5.9	33	7.8	57	6.9
	Neither agree nor disagree	113	27.6	140	33.2	253	30.4
	Mostly agree	214	52.2	151	35.8	365	43.9
	Strongly agree	49	12	91	21.6	140	16.8
	Total	410	100	422	100	832	100
Greater alignment of CAM methods with personal beliefs and values.	Strongly disagree	8	2	10	2.4	18	2.2
	Mostly disagree	39	9.5	21	5	60	7.2
	Neither agree nor disagree	172	42	171	40.5	343	41.2
	Mostly agree	149	36.3	141	33.4	290	34.9
	Strongly agree	42	10.2	79	18.7	121	14.5
	Total	410	100	422	100	832	100
Influence of media and advertisements.	Strongly disagree	42	10.2	20	4.7	62	7.5
	Mostly disagree	117	28.5	46	10.9	163	19.6
	Neither agree nor disagree	158	38.5	142	33.6	300	36.1
	Mostly agree	65	15.9	142	33.6	207	24.9
	Strongly agree	28	6.8	72	17.1	100	12
	Total	410	100	422	100	832	100
In certain stages of illness, patients grasp any hope regardless of the lack of evidence for effectiveness.	Strongly disagree	10	2.4	2	0.5	12	1.4
	Mostly disagree	10	2.4	12	2.8	22	2.6
	Neither agree nor disagree	50	12.2	54	12.8	104	12.5
	Mostly agree	193	47.1	139	32.9	332	39.9
	Strongly agree	147	35.9	215	50.9	362	43.5
	Total	410	100	422	100	832	100

**Table 10:** Reasons for using CAM analyzed by Tukey’s test.

	(I) Substrata	(J) Substrata	Mean difference (I-J)	Std. Error	Sig.	95% Confidence interval	
						Lower bound	Upper bound
Conventional medicine is overly based on technology.	Physicians	Nurses	−0.393^*^	0.121	0.003	−0.68	−0.11
		Patients	−0.549^*^	0.118	0.000	−0.83	−0.27
	Nurses	Physicians	0.393^*^	0.121	0.003	0.11	0.68
		Patients	−0.157	0.079	0.114	−0.34	0.03
	Patients	Physicians	0.549^*^	0.118	0.000	0.27	0.83
		Nurses	0.157	0.079	0.114	−0.03	0.34
Conventional medicine does not treat the person as a whole.	Physicians	Nurses	−0.661^*^	0.132	0.000	−0.97	−0.35
		Patients	−0.770^*^	0.128	0.000	−1.07	−0.47
	Nurses	Physicians	0.661^*^	0.132	0.000	0.35	0.97
		Patients	−0.109	0.086	0.411	−0.31	0.09
	Patients	Physicians	0.770^*^	0.128	0.000	0.47	1.07
		Nurses	0.109	0.086	0.411	−0.09	0.31
Dissatisfaction with the therapeutic capabilities of conventional medicine (treatment outcomes, drug side effects, helplessness in terminal stages of disease).	Physicians	Nurses	−0.367^*^	0.121	0.007	−0.65	−0.08
		Patients	0.105	0.118	0.645	−0.17	0.38
	Nurses	Physicians	0.367^*^	0.121	0.007	0.08	0.65
		Patients	0.472^*^	0.079	0.000	0.29	0.66
	Patients	Physicians	−0.105	0.118	0.645	−0.38	0.17
		Nurses	−0.472^*^	0.079	0.000	−0.66	−0.29
Dissatisfaction with the attitude of doctors and healthcare workers in conventional medicine toward patients.	Physicians	Nurses	−0.627^*^	0.124	0.000	−0.92	−0.34
		Patients	0.132	0.121	0.517	−0.15	0.42
	Nurses	Physicians	0.627^*^	0.124	0.000	0.34	0.92
		Patients	0.759^*^	0.081	0.000	0.57	0.95
	Patients	Physicians	−0.132	0.121	0.517	−0.42	0.15
		Nurses	−0.759^*^	0.081	0.000	−0.95	−0.57
Dissatisfaction with the healthcare system (waiting lists, high costs, lack of all medications covered by the Croatian Health Insurance Fund…).	Physicians	Nurses	−0.703^*^	0.120	0.000	−0.98	−0.42
		Patients	−0.199	0.117	0.203	−0.47	0.07
	Nurses	Physicians	0.703^*^	0.120	0.000	0.42	0.98
		Patients	0.504^*^	0.078	0.000	0.32	0.69
	Patients	Physicians	0.199	0.117	0.203	−0.07	0.47
		Nurses	−0.504^*^	0.078	0.000	−0.69	−0.32
The belief that CAM will help where conventional medicine can no longer assist.	Physicians	Nurses	−0.311^*^	0.108	0.012	−0.57	−0.06
		Patients	−0.227	0.105	0.081	−0.47	0.02
	Nurses	Physicians	0.311^*^	0.108	0.012	0.06	0.57
		Patients	0.084	0.070	0.454	−0.08	0.25
	Patients	Physicians	0.227	0.105	0.081	−0.02	0.47
		Nurses	−0.084	0.070	0.454	−0.25	0.08
Fear of side effects from drugs and therapeutic procedures.	Physicians	Nurses	−0.360^*^	0.111	0.004	−0.62	−0.10
		Patients	−0.390^*^	0.108	0.001	−0.64	−0.14
	Nurses	Physicians	0.360^*^	0.111	0.004	0.10	0.62
		Patients	−0.030	0.072	0.912	−0.20	0.14
	Patients	Physicians	0.390^*^	0.108	0.001	0.14	0.64
		Nurses	0.030	0.072	0.912	−0.14	0.20
Desire for the patient to take a more active role in their treatment.	Physicians	Nurses	−0.707^*^	0.101	0.000	−0.94	−0.47
		Patients	−0.512^*^	0.098	0.000	−0.74	−0.28
	Nurses	Physicians	0.707^*^	0.101	0.000	0.47	0.94
		Patients	0.195^*^	0.066	0.008	0.04	0.35
	Patients	Physicians	0.512^*^	0.098	0.000	0.28	0.74
		Nurses	−0.195^*^	0.066	0.008	−0.35	−0.04
Greater alignment of CAM methods with personal beliefs and values.	Physicians	Nurses	−0.568^*^	0.101	0.000	−0.81	−0.33
		Patients	−0.253^*^	0.098	0.028	−0.48	−0.02
	Nurses	Physicians	0.568^*^	0.101	0.000	0.33	0.81
		Patients	0.315^*^	0.066	0.000	0.16	0.47
	Patients	Physicians	0.253^*^	0.098	0.028	0.02	0.48
		Nurses	−0.315^*^	0.066	0.000	−0.47	−0.16
Influence of media and advertisements.	Physicians	Nurses	0.151	0.120	0.419	−0.13	0.43
		Patients	0.785^*^	0.117	0.000	0.51	1.06
	Nurses	Physicians	−0.151	0.120	0.419	−0.43	0.13
		Patients	0.634^*^	0.078	0.000	0.45	0.82
	Patients	Physicians	−0.785^*^	0.117	0.000	−1.06	−0.51
		Nurses	−0.634^*^	0.078	0.000	−0.82	−0.45
Patients grasp at any hope in certain stages of illness regardless of the lack of evidence for effectiveness.	Physicians	Nurses	0.008	0.099	0.996	−0.22	0.24
		Patients	0.208	0.096	0.078	−0.02	0.43
	Nurses	Physicians	−0.008	0.099	0.996	−0.24	0.22
		Patients	0.200^*^	0.064	0.005	0.05	0.35
	Patients	Physicians	−0.208	0.096	0.078	−0.43	0.02
		Nurses	−0.200^*^	0.064	0.005	−0.35	−0.05

Patients and healthcare workers (HCWs) showed notable dissatisfaction with aspects of conventional medicine, including its reliance on technology (30.6%), lack of holistic care (40.2%), and limited therapeutic outcomes (47%). Many also expressed concerns about the healthcare system’s inefficiencies, such as long waiting times and costs, with higher dissatisfaction among HCWs (52.8%).

A majority (65.2%) believed that CAM offers solutions where conventional medicine falls short, with 58% emphasizing the importance of patient-centered care. Fear of side effects from conventional treatments (52.2%) and alignment with personal beliefs and values (49.4%) further contributed to the appeal of CAM. Media influence was a factor for some (36.9%), while emotional needs, particularly in critical illness stages, led 83.4% to turn to CAM as a source of hope, regardless of evidence (Tables 11–13).

**Table 11:** Cronbach’s alpha coefficient for attitude components.

Attitude components	Cronbach’s alpha	Number of items
Cognitive	0.948	30
Behavioral	0.825	13
Emotional	0.836	13

**Table 12:** Relationship of sociodemographic characteristics to attitude components.

			N	Mean rank	Sum of ranks	Mann–Whitney U	Wilcoxon W	Z	Asymp. Sig. (2-tailed)
Strata	Behavioral	Patients	410	426.16	174,727.5	82,547.5	171,800.5	−1.145	0.252
		HCW	422	407.11	171,800.5				
		Total	832						
	Emotional	Patients	410	434.78	178,258	79,017.0	168,270.0	−2.164	**0.030**
		HCW	422	398.74	168,270.0				
		Total	832						
	Cognitive	Patients	410	475.92	195,128.5	62,146.5	151,399.5	−7.031	**0.000**
		HCW	422	358.77	151,399.5				
		Total	832						
Sex	Behavioral	Male	245	349.27	85,570.5	55,435.5	85,570.5	−5.219	**0.000**
		Female	587	444.56	260,957.5				
		Total	832						
	Emotional	Male	245	354.70	86,902.0	56,767.0	86,902.0	−4.796	**0.000**
		Female	587	442.29	259,626.0				
		Total	832						
	Cognitive	Male	245	380.64	93,256.5	63,121.5	93,256.5	−2.781	**0.005**
		Female	587	431.47	253,271.5				
		Total	832						
Marital status	Behavioral	Married/in relationship	585	426.08	249,254.5	66,645.5	97,273.5	−1.771	0.077
		Single/Divorced/widowed	247	393.82	97,273.5				
		Total	832						
	Emotional	Married/in relationship	585	421.39	246,510.5	69,389.5	100,017.5	−0.903	0.366
		Single/Divorced/widowed	247	404.93	100,017.5				
		Total	832						
	Cognitive	Married/in relationship	585	422.58	247,208.0	68,692.0	99,320.0	−1.123	0.261
		Single/Divorced/widowed	247	402.11	99,320.0				
		Total	832						
Occupation (HCW)	Behavioral	Physicians	100	125.88	12,587.5	7,537.5	12,587.5	−8.019	**0.000**
		Nurses	321	237.52	76,243.5				
		Total	421						
	Emotional	Physicians	100	105.24	10,523.5	5,473.5	10,523.5	−9.962	**0.000**
		Nurses	321	243.95	78,307.5				
		Total	421						
	Cognitive	Physicians	100	104.50	10,449.5	5,399.5	10,449.5	−10.026	**0.000**
		Nurses	321	244.18	78,381.5				
		Total	421						
Employment in oncology	Behavioral	Yes	127	196.01	24,893.0	16,765.0	24,893.0	−1.301	0.193
		No	287	212.59	61,012.0				
		Total	414						
	Emotional	Yes	127	187.46	23,807.0	15,679.0	23,807.0	−2.269	**0.023**
		No	287	216.37	62,098.0				
		Total	414						
	Cognitive	Yes	127	183.88	23,352.5	15,224.5	23,352.5	−2.672	**0.008**
		No	287	217.95	62,552.5				
		Total	414						
Area of residence	Behavioral	Urban	600	415.57	249,339.5	69,039.5	249,339.5	−0.181	0.857
		Rural	232	418.92	97,188.5				
		Total	832						
	Emotional	Urban	600	419.48	251,685.5	67,814.5	94,842.5	−0.575	0.565
		Rural	232	408.8	94,842.5				
		Total	832						
	Cognitive	Urban	600	411.95	247,172.5	66,872.5	247,172.5	−0.878	0.380
		Rural	232	428.26	99,355.5				
		Total	832						

*p* = 0.000 is typically rounded from a very small *p*-value (e.g., <0.001) and does not literally mean zero. It indicates a statistically significant result at conventional thresholds.

**Table 13:** Relationship of sociodemographic characteristics to attitude components.

			N	Mean rank	Kruskal-Wallis H	df	Asymp. Sig.
Age	Behavioral	<20 y	1	450.50	14.582	5	**0.012**
		20–30 y	107	349.00			
		31–40 y	118	462.46			
		41–50 y	206	435.22			
		51–60 y	210	404.67			
		>60 y	190	418.58			
		Total	832				
	Emotional	<20 y	1	716.00	12.528	5	**0.028**
		20–30 y	107	378.92			
		31–40 y	118	450.34			
		41–50 y	206	445.47			
		51–60 y	210	387.78			
		>60 y	190	415.41			
		Total	832				
	Cognitive	<20 y	1	610.50	31.410	5	**0.000**
		20–30 y	107	311.36			
		31–40 y	118	394.44			
		41–50 y	206	443.46			
		51–60 y	210	414.39			
		>60 y	190	461.49			
		Total	832				
Education level	Behavioral	Sec. school	338	392.18	15.362	2	**0.000**
		Bach. degr.	185	472.10			
		Msn. degr.	300	397.27			
		Total	823				
	Emotional	Sec. school	338	401.28	29.569	2	**0.000**
		Bach. degr.	185	492.66			
		Msn. degr.	300	374.34			
		Total	823				
	Cognitive	Sec. school	338	422.05	15.196	2	**0.001**
		Bach. degr.	185	456.74			
		Msn. degr.	300	373.10			
		Total	823				
Work experience	Behavioral	<5 y	67	176.29	10.632	4	**0.031**
		5–15 y	105	213.31			
		16–25 y	114	209.66			
		26–35 y	100	219.72			
		>35 y	36	254.75			
		Total	422				
	Emotional	<5 y	67	179.91	11.334	4	**0.023**
		5–15 y	105	226.16			
		16–25 y	114	204.12			
		26–35 y	100	209.55			
		>35 y	36	256.32			
		Total	422				
	Cognitive	<5 y	67	161.94	21.580	4	**0.000**
		5–15 y	105	216.48			
		16–25 y	114	207.02			
		26–35 y	100	222.12			
		>35 y	36	273.90			
		Total	422				
Religious affiliation	Behavioral	Religious	678	425.65	8.399	2	**0.015**
		Agnostic	118	394.95			
		Atheist	36	314.79			
		Total	832				
	Emotional	Religious	678	422.32	4.213	2	0.122
		Agnostic	118	406.24			
		Atheist	36	340.58			
		Total	832				
	Cognitive	Religious	678	419.80	4.302	2	0.116
		Agnostic	118	422.31			
		Atheist	36	335.36			
		Total	832				
Income level	Behavioral	<1,000 €	238	415.04	6.675	2	**0.036**
		1,000 €	312	441.25			
		>1,000 €	282	390.34			
		Total	832				
	Emotional	<1,000 €	238	413.91	14.134	2	**0.001**
		1,000 €	312	452.70			
		>1,000 €	282	378.64			
		Total	832				
	Cognitive	<1,000 €	238	451.89	17.699	2	**0.000**
		1,000 €	312	432.65			
		>1,000 €	282	368.77			
		Total	832				

*p* = 0.000 is typically rounded from a very small *p*-value (e.g., <0.001) and does not literally mean zero. It indicates a statistically significant result at conventional thresholds.

These findings underscore the emotional, practical, and philosophical drivers of CAM adoption, highlighting the need for integrative approaches in healthcare.

The analysis reveals distinct attitudes toward conventional medicine and CAM among physicians, nurses, and patients. Physicians were less critical of conventional medicine, showing significantly lower agreement than nurses and patients that it is overly based on technology (−0.393 and −0.549, respectively) or fails to treat the person holistically (−0.661 and −0.770).

Nurses expressed more dissatisfaction with therapeutic capabilities and attitudes in conventional medicine compared to physicians (0.367 and 0.627). Patients were even more dissatisfied than nurses regarding provider attitudes (0.759). Fear of drug side effects was higher among patients compared to physicians (0.390), and nurses also reported greater concern than physicians (0.360).

Patients and nurses showed a stronger desire for active patient involvement in treatment compared to physicians (0.512 and 0.195, respectively). CAM’s alignment with personal beliefs was valued more by nurses than physicians (0.568) and by patients compared to physicians (0.315). Patients were also more influenced by media and advertisements promoting CAM than both physicians (0.785) and nurses (0.634). In terminal illness, nurses were more likely than physicians to note that patients grasp at any hope, even without evidence (0.200).

These results highlight differing perspectives on healthcare and CAM, emphasizing the importance of tailored, integrative approaches to address these attitudes.

### 3.6 Cronbach’s alpha coefficient

A comprehensive analysis of the reliability of the measurements, specifically the internal consistency of the particles, was carried out. The internal consistency coefficient, a generalized form that measures the measuring instrument’s internal consistency, was calculated. This coefficient, measured by Cronbach’s Alpha reliability coefficient, provides a thorough and valid understanding of the reliability of the measurements.

The reliability analysis for the attitude components reveals strong internal consistency across all dimensions. The cognitive component shows excellent reliability with a Cronbach’s Alpha of 0.948 across 30 items, indicating a high level of agreement among responses related to beliefs and knowledge about CAM. The behavioral component demonstrates good reliability with a Cronbach’s Alpha of 0.825 across 13 items, reflecting consistent patterns in reported behaviors or intentions to act regarding CAM. Similarly, the emotional component also exhibits good reliability, with a Cronbach’s Alpha of 0.836 across 13 items, capturing stable emotional responses and attitudes toward CAM.

These results confirm that the measures for all three components of attitudes—cognitive, behavioral, and emotional—are robust and suitable for further analysis.

The group of statements describing the cognitive component of the attitude includes the following statements:

“CAM should be integrated with the methods of classical, official medicine”; “Treatment with CAM should be fully covered by Croatian Insurance Fund”; “CAM therapies should be available to patients at the level of primary health care”; “CAM includes ideas and methods whose integration into the system of classical medicine can benefit everyone”; “Clinical medicine should integrate the best of CAM and classical medicine”; “Healthcare workers should be trained to talk with patients about the most frequently applied methods of CAM”; “I know the difference between complementary and alternative medicine”; “During education through verified curricula, healthcare workers should be educated in the field of CAM”; “Health workers should have formal education in the field of CAM”; “During their formal education, healthcare professionals receive very little or no information about CAM”; “Health care professionals do not have enough knowledge to be able to talk with patients about the possibilities of application and effectiveness of CAM”;

“Education in the field of CAM should be an integral part of educational plans and programs for all members of health professions”; “CAM education should be systematically integrated into various classical health contents (from anatomy to internal medicine and health care) at all levels of education, both theoretically and practically”; “Persons who apply CAM and are not health professionals are ordinary charlatans and should be banned from working (R)”; “CAM therapists should go through the licensing system like health workers of classical medicine”; “There should be specialization in CAM therapy”; “CAM therapy should only be practiced by doctors (R)”; “The state should determine who may practice and provide CAM”; “Physical and mental health is maintained by internal energy or life force”; “Health and illness are a reflection of the balance between life-enhancing and destructive forces”; “The body is self-healing and the task of the health worker is only to assist in the healing process”;

“The patient’s symptoms must be considered indicative of a general imbalance or dysfunction affecting the entire body”; “The patient’s expectations and attitudes must be integrated into the health care process”; “Complementary and alternative methods are a threat to public health (R)”; “Therapies that have not been tested according to scientific principles must be banned (R)”; “The effects of CAM therapy are most often the result of a placebo effect (R)”; “CAM therapies include ideas and methods from which classical medicine can profit”; “Most CAM therapies stimulate the body’s natural healing powers”; “Answers to questions in medicine that we do not know the answer to today lie in the folk tradition and knowledge of our ancestors”; “Attitude that CAM will help where classical medicine can no longer help.”

The following statements are included in the group of statements that describe the behavioral component of the attitude (the ability to act toward the object of the attitude):

“Although they do not talk about it, patients in practice use some of the therapies from the field of CAM”; “Among doctors there is strong resistance to the use of CAM in patients who are involved in diagnostic and therapeutic processes”; “Records should be kept on the application of CAM in the patient’s health record/documentation”; “When taking an anamnesis, anamnestic data on CAM applications should be taken”; “The patient must inform his medical team about the application of CAM”; “Patients should consult their doctor or therapist before using CAM”; “I would like that, through formal educational programs, healthcare professionals acquire enough knowledge to be able to have a qualified discussion with interested patients about the possibilities of application and effectiveness of CAM”; “CAM therapy is in principle dangerous for the patient and should be avoided (R)”; “I believe in the Divine/Higher Power and its healing powers”; “The desire for the patient to take a more active role in his treatment”; “Greater compatibility of CAM methods with personal life attitudes”; “Influence of media and advertising”; “In the stages of the disease, patients cling to every hope regardless of the lack of evidence of effectiveness”;

The group of statements describing the emotional component of the attitude (feelings toward the object of the attitude) includes the following statements:

“I am happy that there are complementary and alternative treatment methods”; “I am sad that there is too little talk about CAM methods and that they are rarely used”; “I am surprised by people who do not understand that the motive of CAM therapists is to make money and not the welfare of patients (R)”; “I am proud of those health workers and patients who can openly discuss the benefits and dangers of using CAM”; “I would like it if health workers had formal education from CAM”; “It angers me when people use CAM methods believing in their effectiveness (R)”; “Health workers (doctors, nurses…) are ashamed to talk about CAM with their colleagues”; “I am excited by the thought of the possibilities that may be hidden in CAM methods”;

A test of the difference in the observed factors concerning the observed indicators was carried out. We used the Mann–Whitney U test and the Kruskal–Wallis’s test, two non-parametric statistical tests, to determine whether there are statistically significant differences in the attitudes observed for each of the three components of the attitude for individual sociodemographic characteristics of the respondents. These tests are particularly useful when the data does not meet the assumptions of normality and homogeneity of variance, which is often the case in social science research.

The analysis of attitudes toward CAM revealed significant differences across certain demographic and professional categories based on strata, sex, marital status, occupation, oncology employment, and area of residence, supported by specific mean rank values, while others showed no substantial variation. The findings highlight the stronger emotional and cognitive alignment with CAM among patients compared to HCWs, with significant differences in mean ranks across these groups. Women consistently displayed higher scores across all attitude components. Among HCWs, nurses demonstrated significantly more favorable attitudes than physicians, with large differences in mean ranks. Interestingly, HCWs in oncology showed lower emotional and cognitive attitudes, suggesting a cautious approach within their field. In contrast, marital status and area of residence did not significantly influence attitudes, emphasizing the dominant role of professional roles and gender over personal relationships or geographical location.

The analysis explored the impact of demographic and socio-economic variables on attitudes (behavioral, emotional, and cognitive) toward CAM, revealing significant trends and insights across age, education, work experience, religious affiliation, and income levels. The findings suggest that age, education, work experience, religious affiliation, and income significantly influence attitudes toward CAM, particularly for cognitive and behavioral dimensions. Older individuals and those with longer work experience or lower incomes exhibit more favorable cognitive attitudes, while behavioral attitudes are more positive among religious individuals and those with moderate income levels. These results highlight the importance of socio-demographic contexts in shaping CAM-related attitudes.

From the tables shown above, it can be concluded that occupation, gender, age, level of education, material income, length of service for all respondents in all three attitude components, work in oncology in the emotional and cognitive components, and religious commitment in the behavioral component are factors which lead to statistically significant differences in attitudes. At the same time, area of residence and marital status do not affect the incidence of statistically significant differences in attitudes.

## 4 Discussion

This study provides a detailed examination of the use and perception of Complementary and Alternative Medicine among oncology patients and healthcare professionals in Croatia. The findings reveal significant disparities in CAM utilization and attitudes, shaped by both professional roles and sociodemographic factors. These insights underscore the need for targeted educational and policy initiatives to improve the integration of CAM into oncology care.

### 4.1 Prevalence of CAM use and patterns

The study found that 55.6% of oncology patients had used CAM at least once, compared to 32.2% of healthcare professionals, with statistically significant differences observed (*p* < 0.05). This aligns with global findings that patients often seek alternative approaches to alleviate symptoms, manage treatment side effects, and improve overall quality of life. For oncology patients, the appeal of CAM may lie in its perceived holistic and patient-centered nature, particularly in managing chemotherapy- and radiotherapy-induced side effects such as nausea, fatigue, and stress (Alsharif, 2021).

Among healthcare professionals, nurses and technicians exhibited a higher inclination toward CAM use compared to physicians. Specifically, 27.3% of patients reported using CAM daily, compared to only 4.5% of healthcare workers, highlighting a significant gap in engagement and acceptance. These results mirror international studies showing nurses’ more favorable attitudes toward CAM, potentially due to their patient-focused roles, which often emphasize holistic care (Bou-Young et al., 2023). Conversely, physicians’ lower usage rates reflect their concerns about the lack of robust evidence for CAM efficacy and safety, particularly regarding drug-herb interactions (Berretta et al., 2022).

A systematic review of literature from 2002 to 2017 revealed that 52% of medical specialists accepted CAM, with the highest acceptance observed in family medicine, followed by psychiatry and neurology, while the lowest acceptance was noted in surgery. The overall CAM utilization rate was 45%, with the highest usage reported in obstetrics and gynecology, followed by family medicine, psychiatry, and neurology, and the lowest in surgery (Phutrakool and Pongpirul, 2022).

The prevalence of CAM use among healthcare workers in Trinidad and Tobago was notably high, with an overall rate of 82.3%. This includes 92.4% of nurses, 83.3% of pharmacists, 77.1% of other healthcare providers, and 64.9% of doctors. Despite this high use, knowledge of CAM could have been higher, particularly among physicians, with many health professionals reluctant to recommend or refer patients to CAM physicians (Bahall and Legall, 2017).

The level of education plays a significant role in influencing the use CAM among oncology patients and healthcare professionals. Higher educational attainment is associated with increased CAM utilization, likely due to improved access to information and resources, enabling more informed decision-making. The findings of this study indicate that individuals with tertiary education are more likely to adopt CAM methods compared to those with secondary or lower education levels (*p* = 0.004). Similarly, research conducted in Iran highlights a correlation between the educational level of healthcare workers and their use of CAM, with those possessing higher education being more inclined to engage with CAM modalities (Jafari et al., 2021).

Additionally, research has demonstrated that healthcare professionals who have used CAM modalities are more likely to recommend them to their patients, emphasizing the importance of education and training in enhancing CAM knowledge and recommendations (Jafari et al., 2021). An analysis of a study conducted in Italy revealed that higher education was a significant predictor of CAM use, with an odds ratio of 1.96, indicating that patients with higher education levels were nearly twice as likely to use CAM compared to those with lower educational attainment (Berretta et al., 2016).

Educated patients often have better access to information about CAM, which may influence their decision to use these therapies. They may seek CAM as a complementary approach to conventional treatments, driven by a desire for a holistic approach and greater control over their health (Hutten et al., 2022). Higher education levels may also correlate with a more remarkable ability to critically assess the benefits and risks of CAM, leading to more informed, evidence-based decisions about its use (Balneaves et al., 2022).

This study identifies a significant gender difference in favor of women regarding the use of CAM, a well-documented trend in the literature. Women generally exhibit a stronger preference for holistic approaches. For instance, research conducted in Croatia in 2015 highlighted female gender as a predictor of CAM utilization (Kust et al., 2016). Similarly, a study in Saudi Arabia reported that a significantly higher proportion of women (61.8%) used CAM compared to men (40.0%; *p* = 0.001), emphasizing the role of gender in CAM prevalence among oncology patients (Almouaalamy et al., 2023).

A multi-institutional study in the United States (2021–2022) also identified gender as an independent variable influencing CAM use in oncology patients, with 86% of women utilizing CAM compared to 78% of men (*p* < 0.01) (Hutten et al., 2022). Furthermore, a one-year study in New Jersey found that female gender, younger age, and divorced or single marital status were significantly associated with initiating CAM therapy after a cancer diagnosis. Specifically, women were 1.7 times more likely than men to start CAM therapies post-diagnosis (Perlman et al., 2013). This information provides a comprehensive understanding of the role of gender in CAM use, empowering healthcare professionals to consider this factor in their practice.

The findings of this study identify younger age (40–60 years) as a significant predictor of the use of complementary practices. This age difference in CAM use could have implications for the design of CAM education and counseling programs, as younger patients may have different information needs and preferences. Similarly, Kust et al. reported that CAM users had a mean age of 60, compared to 64, among non-users (Kust et al., 2016).

Research conducted in Turkey in 2021 revealed a higher prevalence of CAM use among oncology patients aged 55–64. This study emphasized the critical role of healthcare professionals in assessing and educating cancer patients about CAM use, particularly given its high prevalence among older patients undergoing treatment (Genc and Bulut, 2024). Similarly, a 2020 study in Germany linked younger age (<62 years) to an increased likelihood of CAM use among cancer patients (*p* = 0.02) (Wolf et al., 2022).

This study did not identify marital status, length of service, area of residence, or religious affiliation as predictors of CAM use. Research conducted in Turkey in 2014 also shows that marital status did not have a statistically significant effect on the use of CAM in patients receiving chemotherapy (Doğu et al., 2014), and the same is stated by Dhanoa et al. (2014). Kust et al. state that the probability of using CAM among divorced persons is higher than among those who are married (Kust et al., 2016), while a study conducted in Korea among older adult oncology patients points out that having a spouse is significantly associated with CAM use, which suggests that marital status may influence the likelihood of CAM use, potentially due to spousal support and encouragement (Chang et al., 2024). Genc and Bulut emphasize the importance of marital support in a study conducted in Turkey, where married individuals show a significantly higher probability of using CAM (Genc and Bulut, 2024).

A systematic review of the literature by Alsharif found an association between marital status and the prevalence of CAM use among oncology patients (Alsharif, 2021). Seven studies indicated that married individuals were more likely to use CAM than their unmarried counterparts. However, three studies reported no significant relationship between marital status and CAM utilization. These findings suggest that while marital status may influence CAM use, all studies do not consistently observe the association.

While this research does not explicitly identify religious attitude as a predictor of CAM use, several studies have shown that religious affiliation significantly influences the use of CAM (Pedersen et al., 2013; Heller et al., 2021). Individuals with strong religious or spiritual attitudes are more likely to engage in CAM practices, often seeing them as complementary to their faith. This relationship is observed in different religious contexts and health conditions, indicating a broader pattern of CAM use among religious individuals (Dima-Cozma and Cozma, 2012; Wirgues et al., 2020).

The tendency of healthcare professionals to incorporate CAM varies across specialties, with some fields showing a stronger preference for its integration. This inclination is influenced by factors such as the perceived benefits of CAM, level of knowledge, and specific patient needs. In this study, healthcare professionals outside of oncology were more likely to use CAM, possibly because oncology and hematology professionals are more aware of CAM’s potential risks and benefits, particularly regarding drug-herb interactions. In New Zealand, healthcare professionals in general practice, physiotherapy, and midwifery are particularly inclined to use CAM. Around 25% of general practitioners practice CAM, and 82.3% refer patients to CAM practitioners (Liu et al., 2021). Primary care providers, in particular, tend to be more open to using CAM therapies compared to specialists, likely due to patient demand and their holistic approach to care (Martz et al., 2006).

In contrast to this study, where area of residence is not a predictor of CAM use, a 2012 study in the Philippines found a higher prevalence of CAM use among rural respondents (68.4%) compared to urban respondents (51.5%), suggesting that the type of community significantly influences CAM utilization (Dahilig and Salenga, 2012).

While the income level among oncology patients is not frequently analyzed as a direct predictor of CAM use, several studies have examined factors influencing CAM use in this population. These studies suggest that, although income is not typically documented as a predictor, other demographic factors, such as education level, play a significant role in CAM use, which can indirectly reflect higher income levels (Berretta et al., 2016). This study aligns with previous research indicating that higher-income individuals are more likely to use CAM (Kust et al., 2016; Xu, 2009).

The relationship between healthcare workers’ income and their use of CAM is complex, influenced by various socioeconomic and demographic factors. Although income is a significant determinant of CAM use, it interacts with other factors such as education, cultural background, and access to conventional health care. Evidence suggests that higher income levels may correlate with increased CAM use, but this relationship varies across populations and contexts (Tor-Anyiin et al., 2018).

Self-perception of health plays a crucial role in predicting the use of CAM. Individuals often turn to CAM to take personal responsibility for their health, seek empowerment, and explore holistic approaches to wellness. This behavior is shaped by their perceptions of illness and health and their experiences with conventional medicine. In the United States, approximately 4 in 10 adults used CAM therapies in the previous year, with CAM users being about 1.5 times more likely to rate their health as “better” than the previous year. Additionally, CAM users were more likely to report their health as “excellent” than non-users (Nguyen et al., 2011). This study and others indicate that individuals who consider their health to be excellent or very good are more likely to engage in CAM practices (Alonso Street et al., 2022).

### 4.2 Attitude toward CAM: cognitive, emotional and Behavioral dimensions

Understanding the distinction between behavior and attitudes is crucial for analyzing the use of complementary and alternative medicine (CAM). While behavior refers to specific actions and the frequency of CAM usage, attitudes encompass individuals’ thoughts, feelings, or intentions regarding CAM. The misalignment between these dimensions can reveal intriguing patterns across diverse sociodemographic groups, offering deeper insights into CAM utilization.

This study examined attitudes toward CAM among oncology patients and healthcare professionals, focusing on three critical attitudinal components: behavioral, emotional, and cognitive. The results revealed significant differences in attitudes between patients and healthcare workers and across various sociodemographic variables, including gender, age, educational level, and financial status. Sociodemographic factors are key to understanding behavioral patterns related to CAM use, offering insights into who most commonly uses these methods and why. However, behavior represents only the outward manifestation of deeper motivations and beliefs. To fully comprehend attitudes toward CAM, it is essential to examine the cognitive, emotional, and behavioral dimensions of attitudes, which reveal more complex patterns of decision-making and perception.

One of the key findings of this research is that patients had statistically significantly higher ranks in the emotional and cognitive components of attitude compared to health workers. This suggests that patients may be more emotionally engaged and cognitively oriented toward CAM, which is consistent with literature indicating that patients often seek CAM for emotional reasons, such as pain or stress relief (Bishop and Lewith, 2010). On the other hand, health professionals may express behavioral attitudes that are more focused on the practical aspects of treatment, such as informing patients about available therapies. This is consistent with the findings of Alsharif, who states that health professionals. However, they may be critical of CAM and often provide information about alternative therapies, particularly in the context of patient education and support (Alsharif, 2021).

In this study, women exhibited significantly higher ranks across all three components of attitude toward CAM than men. Generally, women are more likely to use CAM, particularly in the context of chronic illness, where CAM is often utilized to manage symptoms and enhance quality of life (Astin, 1998). Female health professionals may also be more emotionally engaged with CAM, which may be related to their concern for the patient’s emotional state and recovery.

Older participants in our study showed greater engagement in all three components of attitude toward CAM. Older people, faced with chronic diseases and reduced quality of life, often seek alternative treatment approaches that can help alleviate symptoms and provide psychological support (Bishop and Lewith, 2010). This result is consistent with research suggesting that older people use CAM more often, as they may seek alternatives to traditional medical treatments that are not always effective in treating chronic diseases (Alsharif, 2021).

Lower educational level and lower material status are associated with higher ranks in all components of attitude toward CAM. People with less education may have less access to information about the scientific basis of conventional medical methods and, therefore, more easily turn to CAM as an alternative. Also, participants with lower incomes might be more inclined to use CAM because of the lower costs and easier availability of these therapies, especially in countries where the conventional healthcare system is not always accessible. People with lower incomes often use CAM for economic reasons because in some countries it is often more affordable than conventional treatments, and people with lower incomes may turn to alternative therapies that offer them a cheaper or simpler option. Bishop and Lewith state that people with lower incomes may use CAM for financial reasons, but also because of more difficult access to highly specialized health care (Bishop and Lewith, 2010).

No statistically significant differences were observed in marital status and place of residence, indicating that these factors do not significantly influence attitudes toward CAM. While research has not consistently demonstrated a clear relationship between marital status or place of residence and CAM use, some studies suggest that individuals residing in rural areas may have limited access to conventional healthcare, potentially increasing their likelihood of using CAM.

This research highlights a significant gap between the widespread, unsupervised use of CAM by oncology patients and the awareness and attitudes of healthcare professionals. Physicians tend to exhibit more negative attitudes toward CAM than nurses and technicians, with healthcare professionals generally expressing more significant skepticism than oncology patients. Given the potential impact of CAM on treatment efficacy and outcomes, these findings underscore the need for targeted educational programs to bridge knowledge gaps among healthcare professionals, especially physicians.

### 4.3 Recommendations for practice and policy

To address these gaps, targeted educational initiatives are essential for healthcare professionals, particularly physicians, to improve their understanding of CAM’s role, benefits, and limitations. Integrating CAM education into medical and nursing curricula can equip healthcare providers with the knowledge and skills needed to engage in informed discussions with patients. Such initiatives could also foster interdisciplinary collaboration, enabling healthcare teams to develop holistic treatment plans that align with patient preferences.

Evidence-based guidelines are critical for ensuring the safe integration of CAM into oncology care pathways. These guidelines should address safety concerns, including potential drug-herb interactions, and provide clear protocols for incorporating CAM into conventional treatment regimens. Encouraging open dialogue between patients and providers is crucial to fostering trust and ensuring that CAM use is aligned with evidence-based practices.

### 4.4 Future research directions

While this study provides valuable insights, its cross-sectional design limits the ability to draw causal conclusions. Future longitudinal research is needed to explore the long-term effects of CAM on clinical outcomes, patient satisfaction, and quality of life. Additionally, comparative studies across different cultural and healthcare contexts could provide a deeper understanding of the factors driving CAM adoption and inform global best practices for CAM integration in oncology.

This study underscores the complex interplay of sociodemographic factors, professional roles, and patient experiences in shaping attitudes toward CAM. By bridging the knowledge gap among healthcare professionals and fostering collaboration between patients and providers, the healthcare system can adopt a more holistic and patient-centred approach to cancer care. These efforts are critical for improving patient outcomes, enhancing satisfaction, and optimizing the overall quality of oncology care.

### 4.5 Study limitations

While this study provides valuable insights into the use and perception of Complementary and Alternative Medicine (CAM) among oncology patients and healthcare professionals in Croatia, certain limitations should be considered when interpreting the findings.

First, the cross-sectional design of the study captures attitudes and behaviors at a single point in time, which limits the ability to establish causal relationships between variables. Longitudinal studies would be beneficial to explore changes in CAM usage and attitudes over time and their impact on clinical outcomes.

Second, while the sample size was robust and diverse, it was drawn from a single institution, which may limit the generalizability of the findings to other settings. Replicating the study in multiple institutions or regions would help validate these results and provide a broader perspective.

Third, the reliance on self-reported data introduces the possibility of response bias. Participants may have underreported or overreported their CAM usage or attitudes due to social desirability or recall bias. Future studies could incorporate objective measures or triangulate findings with qualitative methods to enhance validity.

Finally, the study primarily focuses on CAM use and attitudes within the Croatian context, where cultural and systemic factors may differ from those in other countries. While this provides valuable regional insights, comparative studies across diverse healthcare systems and populations would help contextualize these findings globally.

Despite these limitations, the study offers critical contributions to understanding CAM use and perceptions in oncology care and lays the groundwork for future research and policy development in this area.

## 5 Conclusion

This study provides critical insights into the use and perception of Complementary and Alternative Medicine (CAM) among oncology patients and healthcare professionals in Croatia, addressing the three primary objectives outlined in the introduction.

First, the study determined the prevalence of CAM use among oncology patients and healthcare professionals. The findings indicate that 55.6% of oncology patients and 32.2% of healthcare professionals have used CAM at least once in their lifetime. Among healthcare professionals, nurses and technicians reported higher CAM usage compared to physicians. These results highlight a significant discrepancy in CAM adoption, with patients being more inclined to seek alternative therapies to complement their conventional treatments.

Second, the study analyzed the cognitive, affective, and behavioral components of attitudes toward CAM among the two groups. Oncology patients expressed more positive attitudes across all three components compared to healthcare professionals. Factors such as gender, age, education level, income, and personal or cultural experiences with CAM influenced these attitudes. Women, older individuals, and those with lower education or income levels showed more favorable perceptions. Among healthcare professionals, those less directly involved in oncology care demonstrated more openness to CAM.

Third, the study explored the relationship between sociodemographic variables, personal experiences with CAM, and expressed attitudes. It was observed that personal experience with CAM strongly correlated with positive attitudes, especially among nurses and technicians. Healthcare providers’ skepticism, particularly among physicians, often stemmed from a lack of education and concerns about potential risks, including interactions with conventional treatments.

These findings underscore the need for targeted educational initiatives to bridge the gap between patient preferences and healthcare providers’ knowledge of CAM. Such programs could enable physicians and other healthcare professionals to engage in informed discussions with patients about CAM, fostering a more collaborative and integrative approach to oncology care. Additionally, evidence-based guidelines should be developed to ensure the safe and effective use of CAM, addressing safety concerns and supporting its integration into clinical practice.

While this study provides valuable data, its cross-sectional design limits the ability to draw causal inferences regarding the impact of CAM use on clinical outcomes. Future longitudinal studies are needed to investigate these effects over time, particularly the long-term implications of CAM integration in oncology care.

In conclusion, this research highlights the critical role of education, communication, and policy development in optimizing CAM use in oncology. By addressing the knowledge gap among healthcare professionals and aligning clinical practices with patient preferences, the healthcare system can embrace a more holistic approach to cancer care. These efforts are essential for improving patient outcomes, satisfaction, and the overall quality of oncology care.

## Data Availability

The raw data supporting the conclusions of this article will be made available by the authors without undue reservation.
